# TOIB Study. Are topical or oral ibuprofen equally effective for the treatment of chronic knee pain presenting in primary care: a randomised controlled trial with patient preference study. [ISRCTN79353052]

**DOI:** 10.1186/1471-2474-6-55

**Published:** 2005-11-07

**Authors:** Pamela L Cross, Deborah Ashby, Geoff Harding, Enid M Hennessy, Louise Letley, Suzanne Parsons, Anne E Spencer, Martin Underwood, the TOIB Study Team

**Affiliations:** 1 Centre for Health Sciences, Barts and The London, Queen Mary's School of Medicine and Dentistry, 2 Newark Street, Whitechapel, London E1 2AT, UK; 2 Medical Research Council General Practice Research Framework, Stephenson House, North Gower St, London NW1 2ND, UK; 3 Wolfson Institute of Preventive Medicine, Barts and The London, Charterhouse Square, London EC1M 6BQ, UK; 4 Department of Economics, Queen Mary's University of London, Mile End Road, London E1 4NS, UK

## Abstract

**Background:**

Many older people have chronic knee pain. Both topical and oral non- steroidal anti-inflammatory drugs (NSAIDs) are commonly used to treat this. Oral NSAIDS are effective, at least in the short term, but can have severe adverse effects. Topical NSAIDs also appear to be effective, at least in the short term. One might expect topical NSAIDs both to be less effective and to have fewer adverse effects than oral NSAIDs. If topical NSAIDs have fewer adverse effects this may outweigh both the reduction in effectiveness and the higher cost of topical compared to oral treatment. Patient preferences may influence the comparative effectiveness of drugs delivered via different routes.

**Methods:**

TOIB is a randomised trial comparing topical and oral ibuprofen, with a parallel patient preference study. We are recruiting people aged 50 or over with chronic knee pain, from 27 MRC General Practice Research Framework practices across the UK. We are seeking to recruit 283 participants to the RCT and 379 to the PPS. Participants will be followed up for up to two years (with the majority reaching one year). Outcomes will be assessed by postal questionnaire, nurse examination, laboratory tests and medical record searches at one and two years or the end of the study.

**Discussion:**

This study will provide new evidence on the overall costs and benefits of treating chronic knee pain with either oral or topical ibuprofen. The use of a patient preference design is unusual, but will allow us to explore how preference influences response to a medication. In addition, it will provide more information on adverse events. This study will provide evidence to inform primary care practitioners, and possibly influence practice.

## Background

Osteoarthritis (OA) is a common condition, particularly in older people[1]. Around 36% of those aged over 50 suffer from knee pain[2-4], half of whom have severe difficulty with physical function or severe pain[5]. The vast majority of patients who seek care receive it in primary care. Since much of this pain is due to OA, and the only treatment convincingly shown to slow progression of OA is surgery, primary care management should target pain and disability[6]. Analgesics and non steroidal anti-inflammatory drugs (NSAIDs) are the most commonly prescribed drugs for knee pain in older people.

### NSAIDs for knee pain

Oral NSAIDs reduce pain in those with knee osteoarthritis[7]. Despite the risks of gastro-intestinal side effects, renal insufficiency, hepatic toxicity, exacerbation of asthma, sodium retention, raised blood pressure and resistance to anti-hypertensive drugs[8], oral NSAIDs are widely used for the symptomatic treatment of OA in older people[9]. In 2003 over 20 million prescriptions for oral NSAIDs, at a cost of over £250 million, were dispensed in England[10]. There are few data on the direct, indirect and intangible costs and cost offsets from using NSAIDs in older people; the personal and economic costs of managing adverse effects are, however, large. Around 40% of hospital admissions with upper gastrointestinal bleeding, and 40% of associated deaths in older people, are related to NSAID use[11].

### Topical NSAIDs

An alternative to using oral NSAIDs is to use topical NSAIDs, which may have fewer side effects as a result of lower serum concentrations[12]. In 2003, 4.5 million prescriptions for topical antirheumatics were dispensed in England, at a cost of £25 million[10].

There are data to show that topical applications of ibuprofen achieve therapeutic concentrations in deep compartments[13]. Thus they could have pharmacological effects on peri-articular and intra-articular structures, as well as having effects through peripheral and central sensitisation[14]. The continued popularity of rubefacients, with no active ingredient, supports the idea that patients' responses to topical NSAIDs may also be partly mediated through the act of rubbing the affected part and the patients' expectation of receiving a benefit[15].

A meta-analysis of studies using topical NSAIDs concluded that they were more effective than placebo ointments for chronic musculoskeletal disorders at up to two weeks of use[16]. Another meta-analysis considering longer periods of use found that topical NSAIDs were no more effective than placebo at three or four weeks of use[14].

If :

a) the combined effect of NSAID in the ointment, the act of rubbing, and the patients' expectation of benefit produces an effect on pain and disability, **and **

b) topical NSAID preparations have fewer adverse effects compared to oral preparations,

then topical preparations may be preferable to oral ones as routine treatment for older patients with knee OA, as there will be fewer side effects in those whose pain can be managed effectively by topical NSAIDs.

### Choice of NSAID and chronic knee pain to study

There are compelling reasons for choosing ibuprofen to treat chronic knee pain when comparing topical and oral NSAIDs:

• different NSAIDs appear to be equally effective in the treatment of knee OA[17].

• a meta-analysis of the risk of gastrointestinal side effects found that low-dose ibuprofen had the lowest risk compared to other NSAIDs [18].

• the reduction in risk of gastrointestinal side effects is similar when comparing high-dose ibuprofen with either low-dose oral ibuprofen or with Cox-II inhibitors[19]. Ibuprofen is widely used both orally and topically for the treatment of osteoarthritis. In 2003 there were five million and one million prescriptions issued for oral and topical ibuprofen respectively, in England. These represent 25% of oral and 22% of topical NSAID prescriptions.

• most chronic knee pain is thought to be secondary to osteoarthritis[20]. There are problems in diagnosing OA, in that many older people have x-ray changes of OA without experiencing symptoms, and even when x-ray changes are present, OA may not be the cause of their pain.

• x-ray evidence of OA has little impact on pragmatic general practice management of knee pain in older people; indeed, most patients are treated without any x-rays being taken.

### Objective of study

The main objective of this study is to evaluate the benefits and risks of oral and topical ibuprofen in older people with chronic knee pain. A secondary objective is to explore patients' attitudes to medication for knee pain.

### Health economic objectives

We will look at the cost effectiveness of topical and oral ibuprofen in terms of three key research questions:

1. What are the societal costs and benefits of implementing the programme, in terms of the impact the programme has upon the NHS, patient and other service providers?

2. What is the cost effectiveness of the programme over a one-year period and how is this influenced by treatment compliance?

3. What is the predicted long-term cost effectiveness of the programme based on the likelihood and extent of major and minor side effects?

## Methods

The randomised controlled trial (RCT) will evaluate, for people with chronic knee pain, the difference in effectiveness (primarily at one year), and the difference in side effects from general practitioner treatment with oral versus topical ibuprofen over the follow up period. As this is a study lasting over a year, we expect that many patients will change their treatment if pain relief is inadequate. If they do need to change, they are asked to keep to the same route, oral or topical, if possible. For this reason, we expect that the pain outcome at one year will reflect some, but not all, of the difference in effectiveness of the treatments.

Consequently this study has elements of both a difference study and an equivalence study[21]. Our hypothesis is that the oral treatment will have greater side effects. Patients for whom the oral and topical treatment would be equally effective at pain relief will be expected to have more side effects if they are allocated to oral rather than topical treatment. Patients who persist with topical treatment that is less effective than oral treatment may have more pain and fewer side effects. Patients who rapidly change if topical is less effective than oral are likely over the longer term to have similar side effects and pain to patients already on oral treatment. Because patients cannot be expected to remain on inadequate treatment, an intention-to-treat analysis is more appropriate than an on-treatment analysis. The latter would be required for a typical equivalence study. Understanding the results depends crucially on the pattern of combined benefits and harms from the two treatments. However, as the combined effects require a judgement to be made of the relative value of pain relief and side effects, we will first analyse pain and side effects separately. For pain we will be considering whether the outcomes are different or equivalent; for side effects we are interested only if they are different. Then we will seek to demonstrate whether overall patient outcomes (benefits and harms) are better, or worse, if general practitioners advise treatment with either topical or oral NSAIDs, for a range of weightings of pain and side effects.

### Patient preference study

In addition to the RCT, there is a parallel patient preference study (PPS). This will enhance the external validity of the study because:

a) we can establish whether strong preferences affect the relative outcomes. The results of RCTs may not be generalisable if those with strong preferences for a particular treatment are excluded[22].

b) the difficulties of recruiting trial participants from primary care are well known. Allowing those who have a preference for one treatment to be recruited to a PPS will provide more observational data.

c) an RCT would have to be very large to identify any differences in serious adverse events. Including data collected from the PPS will provide further information.

Additionally we will:

a) explore study participants' perceptions of treatment using depth interviews with a theoretical sample of participants. This integration of qualitative data into the interpretation of the quantitative data may provide insights into any unexpected or anomalous findings[23,24].

b) collect information on treatment preferences prior to randomisation.

### Participant inclusion and exclusion criteria

The inclusion and exclusion criteria are as follows:

#### Inclusion criteria

• aged 50 or over.

• have ever had pain in or around the knee on most days for at least a month **and **have experienced knee pain for more than three months out of the preceding year.

• GP consultation, or treatment, for knee pain in the preceding three years.

• informed consent.

• agreement to use chosen or allocated treatment.

• GP agreement to prescribe oral/topical ibuprofen.

• ability to complete postal questionnaires.

#### Exclusion criteria

• peptic ulceration (past or current).

• current moderate or severe indigestion.

• previous severe adverse reaction to NSAIDs.

• hypertension (systolic BP of 155 mm of Hg or more **or **a diastolic BP of 105 mm of Hg or more).

• uncontrolled heart failure.

• creatinine > 140 mmol/L.

• abnormal liver function sufficient to contraindicate use of NSAIDs (as liver function tests performed and reference ranges vary between different laboratories, this decision is at the discretion of the participant's GP).

• GP request not to include.

• serious psychological or psychiatric disorders (including dementia).

• previous knee replacement/s or awaiting knee surgery.

• inflammatory arthropathy.

• pain referred from hip or back.

• serious injury within six months.

• currently on anticoagulants or oral steroids.

• anaemia (Hb <12.4 g/L for men or <11.8 g/L for women).

• disseminated malignancy.

To meet the American College of Rheumatologists' (ACR) clinical criteria for osteoarthritis of the knee, patients need to have knee pain, as defined for this study, and meet three out of the following six criteria [25]:

• aged over fifty.

• less than 30 minutes morning stiffness.

• crepitus.

• bony tenderness.

• bony enlargement.

• no palpable warmth.

Measuring the proportion of our sample meeting each of these criteria will allow us to describe our sample more accurately, and assess whether any of these criteria affect outcome.

### Participant identification and recruitment

#### Location

The study is taking place in 27 practices (plus two pilot practices) from the Medical Research Council General Practice Research Framework GPRF[26]. We sought to select practices that were nationally representative in terms of region, deprivation and type of locality (inner city/urban/suburban/rural).

#### Identifying potential participants

In order to maximise recruitment we are using three approaches to identify potential participants:

a) searching electronic medical records within general practices for patients aged 50 or over who have consulted with OA or knee/leg pain in the preceding 5 years.

b) searching electronic prescribing databases for all patients aged 50 or over who have received a prescription for oral/topical NSAIDs or a rubefacient over the preceding year.

c) during the study recruitment period, GPs are asked to notify the practice research nurse when potentially eligible patients consult.

After training, the practice-based research nurses perform a search on the practice computer using MIQUEST[27]. This program, which was obtained from the National Health Service Information Authority, will search nearly all GP software in current use. The search selects patients over 50 who either have a diagnosis of osteoarthritis or knee pain recorded within the last 5 years, or who have received a prescription for NSAIDs or a rubifacient over the last 12 months.

The output from this search generates a comma-delimited file, on a floppy disk, containing the patients' name and address data. A bespoke software program generates study ID numbers, personalised approach letters and participant registers for the research nurse. This program is sent out to practices on a laptop computer, with a printer and pre-printed study paperwork. The nurses use this computer, and the data generated from the practice computer system, to print names and addresses on the invitation letters. After printing, all patient data are removed from the study computer. This approach minimises access to the patient records for research purposes, ensures all patient-identifiable data remains within the practice until explicit consent has been given for it to be released to the study team, and automates the production of study paperwork.

#### Initial approach questionnaire

The list of potential participants is screened by the GP and those whom it would be inappropriate to approach, for example those patients with terminal illness or serious psychological disorders, are removed. Invitations to participate, trial information sheets, questionnaires to screen for eligibility and expression of interest forms are sent from and returned to the practice.

#### Initial assessment

The practice-based research nurse contacts interested patients who, from the initial approach questionnaire, appear eligible. At the initial assessment:

a) the trial is explained to the potential participant.

b) eligibility is confirmed.

For those potential participants who are still eligible and interested:

a) blood pressure and peak expiratory flow rate are measured.

b) blood is collected for full blood count, renal function, liver function and serum ferritin.

c) arrangements are made for a medical assessment prior to a baseline assessment one-two weeks later.

d) potential participants are asked not to use any topical or oral NSAIDs for one week before baseline assessment.

#### Medical assessment

Between the initial and baseline assessments potential participants attend for a brief clinical assessment by a general practitioner, to identify components of the ACR clinical criteria for knee OA. The general practitioner also confirms, in light of the laboratory results, the potential participant's eligibility for the study, and agrees that he or she will be willing to prescribe either oral or topical ibuprofen for this potential participant. A patient with contraindications to either oral or topical ibuprofen cannot enter the study, either in the RCT or the PPS.

#### Baseline assessment

Eligible and interested patients return one to two weeks later to complete baseline questionnaires, to have baseline blood pressure, peak expiratory flow and forced expiratory volume measured, and to complete consent forms. Immediately after baseline assessment those consenting to join the RCT are randomised. All participants are provided with a starter pack of their chosen/allocated treatment when randomised to ensure that they can start treatment immediately.

**Figure 1:**
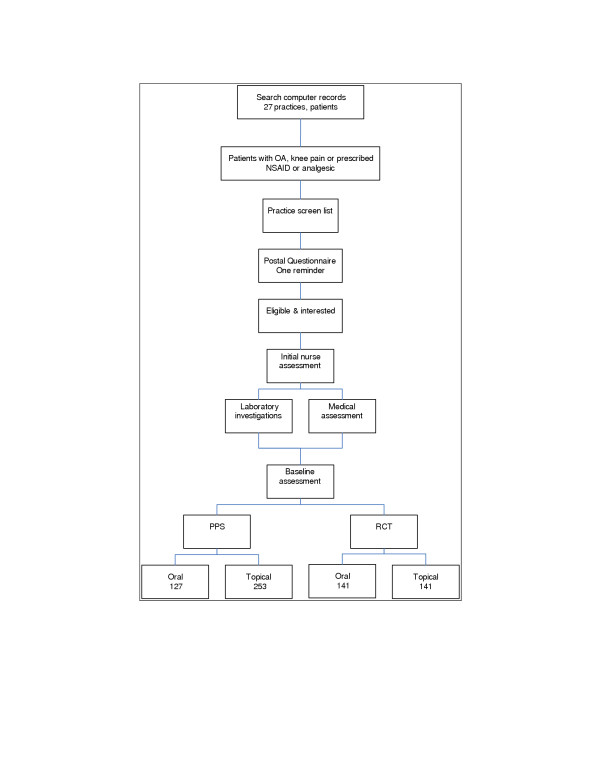


The assessment procedures are the same for patients in the RCT and PPS. Figure 1 shows the recruitment process, with the calculated recruitment targets.

#### Allocation and protection from bias

A remote telephone randomisation service, separate from the main study team, uses computer-based randomisation to register patients joining the study and to allocate RCT participants to treatment groups. Randomisation is stratified by practice, severity of pain, age and source of patient.

The main study team are blind to participants' chosen/allocated treatment. The trial statistician, who is not involved in data collection, has information on chosen/allocated treatment for the data monitoring and ethics committee.

At a practice level the study is not blinded. The main outcome measures are all based on self-completed questionnaires; clinical outcomes are measured at baseline, 1 and 2 years (or end of study if sooner). Blood is analysed in the practice's usual NHS laboratories. Blood pressures are the average of three readings using a Compact Dinamap (Johnson & Johnson). Respiratory function is an average of three readings made using a Clements Clarke one flow tester ATS 94 spirometer. Mortality data are collected from practices and the NHS central registry. Prescribing, selected diagnostic and hospital admission data are collected from patient records by practice nurses.

#### Interventions

The two interventions being compared are the GP's recommendation (either a prescription or advice to get an over-the-counter preparation) to use either topical or oral ibuprofen. For those whose chosen/allocated treatment is oral ibuprofen, practices are asked to use no more than 1.2 g per day. Treatments for knee pain other than NSAIDs may be used as each patient's doctor thinks appropriate.

Adherence with chosen/allocated treatment will be assessed using:

1. a summary of GP prescriptions issued for the trial participants, converted into Average Daily Quantities for topical/oral ibuprofen and other topical/oral NSAIDs.

2. participant self-report of the number of times they have used pain-killing tablets or rubbing ointments in the month previous to each of the questionnaires, plus information in the same questionnaires on whether they have changed treatment during follow-up.

#### Follow-up

**Figure 2:**
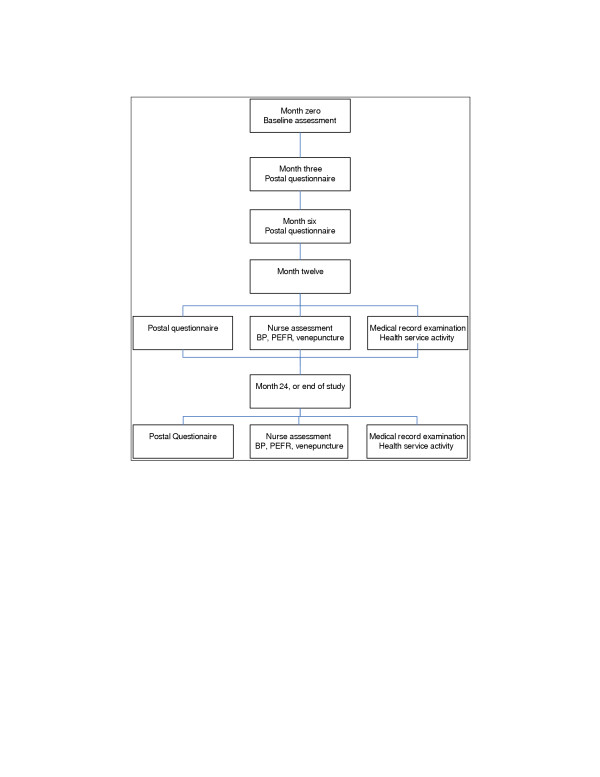


Follow-up is organised centrally. Postal questionnaires consist of the same package of instruments collected at baseline. Participants are sent postal questionnaires three, six, 12 and 24 months after randomisation. One year and two years after randomisation participants are asked to visit the practice to have their blood pressure and respiratory function measured and blood taken for full blood count, serum ferritin, creatinine and liver function tests. The medical records are examined one year after randomisation to identify unplanned hospital admissions, and after two years (or at the end of the study) to collect health service activity data and confirm reported changes in medication and adverse effects. Follow-up procedures are summarised in Figure 2.

We are taking the following steps to keep loss to follow up to a minimum:

1. there are two reminders for each follow up questionnaire, the second by recorded delivery.

2. participants who are unable to attend surgery for annual follow-up will be visited at home by the practice nurse.

3. participants are flagged at NHS central registry to ensure that we identify all deaths, and changes of general practitioner. This will also allow us to locate participants who have moved house for follow-up.

4. participants who have withdrawn from treatment continue to be followed up, with their consent.

#### Qualitative study

Depth interviews will be conducted on a sample of participants during the study. A theoretic sampling strategy will be used to explore the theory that 'older' people's beliefs about the efficacy of topical and oral ibuprofen are shaped by their social role as non-economically productive members of the social order[28]. The theory to be explored postulates that older people do not act as consumers in respect of their use of topical and oral ibuprofen, but rather accept at face value 'expert' knowledge from health professionals.

A theoretic sample will be generated. Informants will be selected based on their age, severity of pain, treatment choice/allocation, and occurrence of adverse events. These interviews will be recorded, transcribed and analysed using the principles of theory- informed qualitative analysis to establish the veracity of the constructed theoretical model[29]. To avoid any possibility that the interviews could, themselves, affect the participants' responses to the main outcome measures, data from these participants will not be included in the main analysis.

### Outcome measures

Data collection is the same for the RCT and PPS. The outcome measures include measures of pain and disability, quality of life, use of medication and adverse events. Health economic data will also be collected.

### Patient pain or quality of health outcomes

#### Primary outcome measure

• the Western Ontario and McMaster Universities Osteoarthritis Index (WOMAC) questionnaire, which measures pain and disability in the preceding 48 hours[30].

#### Secondary outcome measures

• the postal version of the Chronic Pain Grade[31], which measures pain and disability over the preceding six months.

• the EQ5D[32,33], a measure of health-related quality of life.

• the SF-36 version 2[34], a different measure of health related quality of life.

• a question assessing satisfaction with treatment.

#### Major possible adverse effects

The proportion who die or have an unplanned hospital admission will be presented. A trial powered to show a difference in individual major adverse events would be unfeasibly large. For example, in the control arm of a trial of misoprostol for patients taking NSAIDs 1% of patients had a serious upper gastrointestinal complication over six months[35]. The rates of serious gastrointestinal complication in the control groups of the CLASS[36] and VIGOR[37] studies of Cox-2 inhibitors compared with NSAIDs were 0.6% and 1% respectively.

Ascribing causality for individual events to the medication will not usually be possible.

Deaths will be identified by practices when records are withdrawn and by flagging of records at NHS central registry. Unplanned hospital admissions will be identified from patient-completed questionnaires and annual medical record examination. Cause of admission will be ascertained from medical record. If necessary, the unplanned nature of an admission will be confirmed by the practice nurse contacting the participant.

#### Minor possible adverse effects

A composite binary measure of minor adverse events will be developed, consulting with general practitioners using the Delphi technique. We will define these as changes in selected parameters serious enough for a change of treatment to be advised. We will collect data on the following parameters indicative of 'minor side effects'; these will be reported individually, and they may also contribute to the overall composite measure:

• iron deficiency or iron deficiency anaemia. data from the Framingham study show that the prevalence of iron deficiency in NSAID users, measured by serum ferritin (2.7%), is over twice the prevalence of iron deficiency anaemia (1.2%) in a healthy elderly population[38]. Ferritin may therefore be a useful proxy for occult gastrointestinal bleeding.

• new diagnosis of hypertension or failure of existing anti-hypertensive treatment or increase in blood pressure during follow up.

• New diagnosis of asthma/chronic obstructive pulmonary disease (COPD), or a new prescription of either a beta-2 agonist or a steroid inhaler, or a 15% fall in peak flow. Explicit new diagnoses will be identified from the medical record. In a previous GPRF study, recording of new diagnoses of asthma in general practice record was found to be unreliable[39]. For this reason the issue of a beta-2 agonist inhaler to someone who has not had one in the preceding year will be used as an indicator of a new diagnosis of asthma/COPD. Deterioration in asthma/COPD control will be considered present if a patient is initiated on a steroid inhaler. Deterioration in lung function measures will also be used.

• Renal impairment The upper limit of the normal range for creatinine in older people is 160 mmol/L[40]. This is higher than in a younger population. Patients with a creatinine >140 mmol/L at baseline will not be included.

• Heart failure Few GPs have access to echocardiography to confirm the diagnosis of heart failure. For this reason any new diagnosis of heart failure in the practice records will be included.

• Indigestion An increase in recorded indigestion by more that one category on a five-point Likert scale.

#### Compliance

• decision to stop NSAIDs for any reason during the study period.

• use of other analgesics during the study period.

#### Health economic outcomes

The two main outcomes that arise from medication are alleviation of knee pain balanced against the adverse effects of medication. The main HE analysis uses a combined summary measure of quality of life using the EQ-5D and the SF-6D[41]. It is recognised, however, that our summary quality of life measures, taken at different time intervals, may not adequately capture adverse effects from medication due to their fluctuating and temporary nature. Initially we had proposed to model 'any presumed link' between minor and major effects and death. This no longer seems appropriate given that exposure to adverse effects of medication is likely to lead to a discontinuation or switching of medication. We will instead conduct secondary analysis based upon the clinical measures of pain and adverse effects. If the pain levels are similar in the two treatments, and only the adverse effects are statistically different, we will use the composite binary measure of minor adverse events to calculate the 'number needed to harm' for both oral and topical medication[42]. This calculation gives the number of patients who must receive medication before we expect to see one harmful case of adverse effects. If both pain and adverse effects are statistically different between the two medications, we will attempt to combine the measures of pain and adverse effects, informed by patients' preferences for medication based on patients' a) compliance to the regime and b) switching to the alternative medication. This secondary analysis will also draw upon the qualitative study of patients' attitudes towards medication.

#### Costs

Costs will be obtained by recording units of resources, e.g. GP consultations, drug purchases and hospital attendance, used in both groups and applying a tariff to each type of unit. Health service usage will be based upon patients' self-reported usage, validated against medical records. Where possible, local cost tariffs will be used with national sources as a comparator. The costs to participants and their families will be obtained from the patient questionnaire.

The incidence of serious adverse effects caused by ibuprofen is such that a much larger trial is required to identify an important difference between the two groups. However these events may have large financial and other costs. Particular care, therefore, will be made in measuring the financial impacts of side effects.

### Sample size

#### The sample size estimate is based on the primary efficacy measurement at one year

Previous work has shown minimum differences in WOMAC pain and disability scales perceptible to patients are around 10–12 mm on a 100 mm visual analogue scale[43]. Typical standard deviations for the change between baseline and follow-up in knee OA trials are around 22 mm. The results will be presented for the difference between groups in the change from baseline in WOMAC mean score with their 95% confidence intervals. To show a difference of 10 mm with 90% power and 5% significance we need analysable data on 103 subjects in each group. Assuming a 75% follow-up rate at one year, this means we need to recruit 275 participants to the RCT. This will also show equivalence to within 10 mm at 80% power.

Early recruitment data for the PPS indicate a 3:1 preference for topical compared to oral treatment; allowing for this imbalance, we need to recruit 368 participants to the PPS to achieve 90% power.

When the study was first started, it was planned that we would recruit to both RCT and PPS from all participating practices. However early recruitment data indicated that twice as many people would join the PPS when compared to the RCT, suggesting that we would overshoot our PPS recruitment target whilst not reaching the more important RCT recruitment target. For this reason, with the agreement of the funders, the trial steering committee and the data monitoring and ethics committee, we are recruiting to the RCT only in the last seven practices to join the study.

It is usual in equivalence studies to do an on-treatment analysis rather than an intention-to-treat analysis. However, as this study is testing two approaches to managing knee pain, it was agreed that an intention-to-treat analysis would be appropriate, although on-treatment analyses of side effects will also be carried out.

### Analysis

Initially the RCT and PPS will be analysed separately. The primary and secondary outcomes of pain and health status, side effects and compliance (which includes drug use and mode of delivery) will be described and analysed on an intention-to-treat basis. The first analyses will be on outcomes or changes in outcomes at one year. This is the period for which the most substantial amounts of data will be available. There will also be an on-treatment analysis of side effects, which will report results for oral or topical treatment both before and after adjusting for other drug use. Although the data on timing of side effects is limited, it will be possible to produce estimates of the rate of side effects and hazard ratios for the effect of the mode of different treatment as well as for dosage and other pain killers.

The joint distributions of pain at one year and side effects at or by one year will be plotted. The effect of a range of relative utilities of pain and side effects will be incorporated into the analysis to produce more information on the relative advantage of the oral vs topical approach.

Prior to any analysis, and blind as to the treatment arm, we will have checked and validated the data. Rules for classification of information on side effects, hospital admissions and drug usage, each of which can come from a number of sources, will have been set, implemented and checked. Missing data in the health scores will be dealt with as recommended in the relevant manuals.

#### Details of analyses

All results will be presented with 95% confidence intervals.

#### ITT analyses comparing topical and oral treatment

For binary outcomes, the differences in proportions, as well as the odds ratio (from unadjusted logistic regression) will be presented. The effect of any failure of the randomisation will be investigated for the RCT, but adjustment will be made for the expected difference between the groups in the PPS using logistic regression.

For ordinal data with 5 categories or fewer the results will be presented as tables of proportions.

All other scores will be treated as quantitative date and will be analysed using t-tests or multiple regression.

Normally the change in WOMAC score from baseline would be the most appropriate measure, adjusting as it does for baseline pain, but as absolute levels of pain may be more strongly associated with treatment dose, and hence side effects, this measure will be included as well. If the randomisation is successful then both should be unbiased.

Differences in measures of potential side effects will be used preferentially, although results for absolute values may be presented.

#### On-treatment analysis by mode of drug

The information available on drug usage over the year is not complete. Classification of average daily dose will incorporate the three measures – prescriptions over the period, and the measures of drug use over the previous month and two days as indicated in the questionnaires. Estimates will be obtained for the period covered by each questionnaire and over the first year. Where we know a side effect has resulted in a change of medication during the time a questionnaire covers, estimates of drug use prior to the change will be made from the previous questionnaire (if available) and/or prescribing data as appropriate.

The development of a side effect will be analysed using survival analysis methods allowing for changing predictors. As part of this analysis allowance will be made for other painkillers being taken by the participants. The pain killers will already have been classified according to their likely side effects.

On-treatment analysis of WOMAC scores is unlikely accurately to reflect the treatment effect, as patients with inadequate treatment are more likely to have changed treatment.

#### Combined analysis of WOMAC and side effects

The joint distributions of the WOMAC score at one year and side effects will be displayed graphically by study arm. In order to make judgements on the relative overall benefit of each treatment the results of a) the Delphi study into GPs' attitudes to side effects, and b) the qualitative study of patients' attitudes to them will be incorporated as utilities in a Bayesian analysis of the relative benefits for a range of values of the relative importance of pain and side effects.

If there is a similarity in the relationship between pain and side effects for the four groups, the relationship will be modelled.

#### Sensitivity analyses

The potential effect of withdrawals from the study and missing data will be investigated. Best and worst case scenarios will be given.

#### Comparison of RCT and PPS results

The effects of treatment (by intention-to-treat) will be tabulated for the two studies. Appropriate multiple analysis will be performed adjusting for those predictors expected to be different in the two studies (eg age, sex, attitude to treatment and possible troublesomeness of knee at baseline). Tests will be made for potential interaction of type of study with type of treatment.

The combined outcomes of WOMAC and side effects will be investigated graphically in the two studies to see whether the relationship appears to have similar characteristics. If it does, modelling of the relationship to allow for the different baseline characteristics will be attempted.

#### Health economics analysis

The study will estimate the distribution of costs and outcomes of treatment. This will allow us to investigate the probability that the intervention is cost effective and to establish a confidence interval around the cost effectiveness estimate. Given that there is a band of 'uncertainty' around the measure of adverse effects additional sensitivity analysis will be conducted around this measure to see the impact on the cost effectiveness result.

The secondary health economics analysis will be based upon the clinical measures of pain and adverse effects. If the main difference between topical and oral medications is that the former produce fewer adverse effects, we can estimate the cost of preventing adverse effects caused by oral medication. If the cost of prevention is less than the cost of treating one episode of adverse effects, then there is a clear case for preferring the use of topical medication. If the treatment cost is less than the cost of prevention, then the decision to implement topical mediation rests on a view of the value of preventing the pain and suffering from these adverse effects. The value of this prevention will be informed by both the Delphi study into GPs' attitudes to side effects and the qualitative study of patients' attitudes to them. If both pain and adverse effects are statistically different between the two medications, we will gauge patients' preference for the medications looking at a) compliance to the regime and b) switching to the alternative treatment regime. This secondary analysis will also draw upon the qualitative study of patients' attitudes towards medication[41].

## Discussion

Recruitment started in April 2003. By April 2005 we had completed our recruitment with 276 participants in the RCT and 288 participants in the PPS. Early follow-up rates at one year are around 80–85%, suggesting that we will have ample data for our analyses of the RCT. While the PPS is a little underpowered, it will provide important information to the on-treatment analyses of side effects and effects of compliance.

## Competing interests

The author(s) declare that they have no competing interests.

## Authors' contributions

PC drafted the manuscript and carried out the Delphi study. MU is the principal investigator. DA participated in the study design and the analysis plan. EH drafted the analysis plan. GH planned and performed the qualitative study. SP assisted in the qualitative study and contributed to the manuscript. LL participated in the study design and contributed to the manuscript. AE participated in the study design and wrote the health economic sections of the manuscript. All authors read and approved the final manuscript.

## Pre-publication history

The pre-publication history for this paper can be accessed here:


